# Hematologic parameters in coronavirus infection (COVID-19) and their clinical implications

**DOI:** 10.15190/d.2020.14

**Published:** 2020-10-01

**Authors:** Rao Muhammad Waleed, Inbisat Sehar, Waleed Iftikhar, Huma Saeed Khan

**Affiliations:** ^1^ CMH Lahore Medical College and Institute of Dentistry, Lahore, Pakistan; ^2^ California Institute of Behavioral Neurosciences & Psychology, Fairfield, CA 94534, USA

**Keywords:** Coronavirus, COVID-19, pneumonia of unknown origin, cytokine storm, coagulopathy, D-dimer, thrombophilia

## Abstract

Coronaviruses are a class of enveloped RNA viruses that cause infections of the respiratory tract, characterized by fever, tiredness, dry cough, diarrhea, loss of smell or taste, chest pain and shortness of breath. Many patients with mysterious pneumonia were distinguished in December 2019 in Wuhan. The pneumonia of obscure origin was found to be ascribed to a novel coronavirus and described as novel coronavirus pneumonia (NCP). The Chinese authorities initially reported the wave of mysterious pneumonia on December 31st, 2019 and it was declared as an outbreak of international concern on January 30th, 2020. A systematic search of relevant research was conducted, and a total of 58 primary research articles were identified, analyzed, and debated to better understand the hematologic profile in COVID-19 (Coronavirus disease) infection and its clinical implications. All the findings in this article manifest a true impression of the current interpretation of hematological findings of the SARS-COV-2 disease. Pathophysiology of COVID-19 disease can be better interpreted by taking into consideration the hematologic parameters. Clinical implications of the hematologic profile of COVID-19 patients including cytokine storm, coagulation profile, and thrombophilic complications are under-recognized. Therefore, this review focuses on the coagulation profile, cytokine storm, and its treatment options. The role of pre-existing thrombophilia in COVID-19 patients and how it could result in the poor prognosis of the disease is also debated. The recent data suggests that hypercoagulability could be the potential cause of fatalities due to COVID-19. Potential effects of tocilizumab, metronidazole, and ulinastatin in suppressing cytokine storm may help to treat SARS-COV-2 infection. This review also highlights the significance of thrombophilia testing in SARS-CoV-2 patients depending on the clinical features and especially in pregnant women.

## Summary

*1. Introduction*

*2. Methodology*

*3. Hemodynamics*

*4. Cytokine storm*

*5. Treatment of cytokine storm*

- *5.1. Metronidazole *

- *5.2. Tocilizumab*

- *5.3. Ulinastatin*

*6. Coagulation parameters*

*7. Thrombophilic complications in pregnant women*

*8. Preexisting thrombophilic disorders & COVID-19*

- *8.1. Factor V Leiden mutation*

- *8.2. Antiphospholipid syndrome*

- *8.3. Nephrotic syndrome*

*9. Conclusion*

## 1. Introduction

Coronaviruses are a set of enveloped ribonucleic acid viruses, with some of them having the largest genome if compared with other RNA viruses^1^. They are distributed among humans, birds, and other mammals and are known to cause hepatic, neurologic, enteric, and respiratory diseases, ranging in symptoms from mild to severe^2^. They have a high affinity to the respiratory mucosa, thus affecting the respiratory system to a greater extent in comparison to other systems in the body^2^. Four out of the six known coronaviruses cause typical common cold symptoms in immunocompromised individuals and are not considered highly virulent^1^. The other two, i.e. Middle East respiratory syndrome coronavirus (MERS-CoV) and severe acute respiratory syndrome coronavirus (SARS-CoV) are highly virulent and contagious, with high mortality rates^2^. The SARS outbreaks in 2002 and 2003 were attributed to SARS-CoV^3^, while the MERS outbreak in 2012 was caused by MERS-CoV^4^. 

A group of victims with mysterious pneumonia was recorded in Wuhan in December 2019, with an epidemiological relationship to the wholesale market for wild, wet animals and seafood in Wuhan^5^. The mysterious pneumonia was discovered to be attributed to a novel coronavirus and described as novel coronavirus pneumonia (NCP)^6^. NCP was ascribed to severe acute respiratory syndrome coronavirus 2 (SARS-CoV-2)^2^. Angiotensin-converting enzyme-2 (ACE-2) is the receptor used by SARS-CoV-1 and SARS-CoV-2 to gain entry into the cell^7^. The infection proved to be highly contagious; however, with a lower mortality rate but a higher virulence rate than SARS and MERS^8^. The highly contagious and virulent state of the virus caused a worldwide pandemic that is proving challenging to tackle.

**Figure 1:**
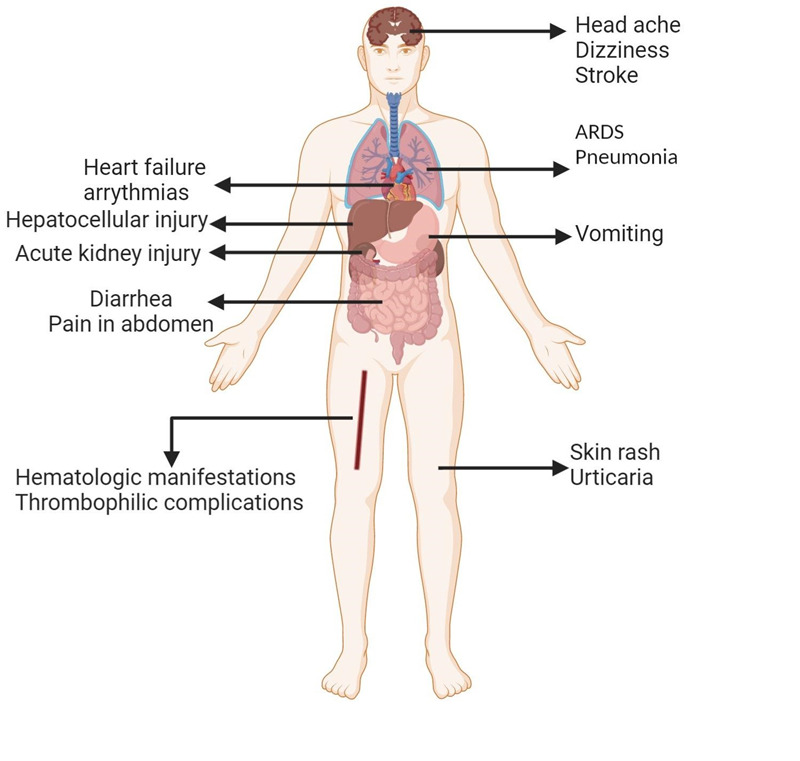
Clinical presentation of COVID-19 patients Adapted from Gupta et al. (2020)^11^. This figure is created using BioRender.com.

With increased research, COVID-19 is now regarded as a significant systemic infection, with its clinical manifestations suggesting the respiratory system, gastrointestinal tract, neurologic system, cardiovascular system, hematologic system, urinary system, and immune system are affected^9,10^. Figure 1 describes the clinical presentation of COVID-19 patients taking into consideration the different body systems.

This paper reviews the hematologic systemic effects with a focus on the hemodynamic picture and parameters that are related to COVID-19, outlining the hemodynamics, coagulation parameters, cytokine storm, thrombophilic implications, and potential clinical outcomes.

## 2. Methodology

An organized review of relevant literature on the hematologic parameters in SARS-CoV-2 infection and its clinical implications was conducted, taking into account case studies, recent journal publications, and current research. The key search terminologies were: COVID-19, cytokine storm, hemodynamics in COVID-19, D-dimer, clinical manifestations, and coagulation. The databases used were Google Scholar, MEDLINE, PubMed, and EBSCOhost. These databases provide an effective and quick method of conducting research, organizing data per subject heading, thus facilitating effective use of keyword search terminologies. For literature to be considered, it had to address COVID-19 and its hematological features, primary sources had to be peer-reviewed and published in credible journals. A total of 100 articles satisfied the criteria for inclusion. Duplicate articles were eliminated, thus remaining with 70 articles. Articles talking on aspects other than COVID-19 and its hematological manifestations, and those that were not peer-reviewed were excluded, further narrowing down the articles to 58.

## 3. Hemodynamics

In COVID-19 patients, hematologic profiles greatly depend on the severity of the disease^12^. The summary of hematologic changes in SARS-COV-2 patients is mentioned in Table 1. A research paper published by Huang et al. (2020)^8^ on the clinical profile of patients infected with SARS-CoV-2 showed normal hemoglobin levels and varying other blood parameters based on the disease intensity^8^. Generally, white blood cell (WBC) count of all patients was below 4×10^9^/L, hence signifying leucopenia. Lymphopenia was also noted with lymphocyte counts of less than 1.0×10^9^/L in 63% of patients^8^. Patients in the intensive care unit (ICU) presented with lower levels of absolute lymphocyte count (ALC). The median nadir ALC of ICU patients was found to be 0.4×10^9^/L, and that of non-ICU patients was 1.2×10^9^/L^8^. A study performed by Liu et al. (2020)^13^ in Changsha, China, incorporating 115 confirmed COVID-19 patients, also identified lymphopenia in most patients, thus agreeing with the study findings of Huang et al. (2020)^8^. Forty-two percent of the study participants having a median age of 42 years were identified with lymphopenia^13^. These lymphopenic patients were observed having other disorders as well, such as leucopenia (48.1% vs 14.8%, P<0.001), eosinophilia (92.6% vs 54.1%, P<0.001), coronary heart disease (3.6% vs 0%, P=0.026), and hypertension (30.8% vs 10%, P=0.006)^13^.**

**Table 1:** Summary of hematologic changes in COVID-19 patients Adapted from Terpos et al. (2020)^12^ with permission.

Parameters	Changes
Thrombocyte count	Decreases
Neutrophil count	Increases
Lymphocyte count	Decreases
Lactate dehydrogenase	Increases
Serum ferritin	Increases
Interleukins (IL-6, IL-2, IL-7)	Increases
C-reactive protein (CRP)	Increases
Procalcitonin	Increases
TNF-α	Increases
Prothrombin time (PT)	Prolonged
D-dimer	Increases
Fibrinogen degradation product (FDP)	Increases

A study done by Fan et al. (2020)^14^ on the hematological manifestations in 69 patients in Singapore, concur with the Chinese study findings of Liu et al. (2020)^13^ and Huang et al. (2020)^8^; with lymphopenia and leucopenia noted among 23% of SARS-CoV-2 patients. Platelet counts were reported to be normal in all studies; however, mild thrombocytopenia was seen in some severe cases of the disease^14^. Neutrophilia was only seen in ICU patients, which developed during hospitalization; however, non-ICU patients tend to have normal neutrophil counts^14^. The cause of neutrophilia is still unknown, whether it is nosocomial in origin or an indication of worsening disease.

Pregnant females infected with SARS-CoV-2 are more susceptible to be affected by lymphopenia. Huanhuan et al. (2020)^15^ study on pregnant women with NCP showed that lymphopenia was more common at a rate of 64% in confirmed pregnant females compared to the non-pregnant group (56%)^15^.**

The occurrence of lymphopenia varies geographically. For example, Fan et al.'s (2020)^14^ study in Singapore identified only 23% of patients having lymphopenia, in comparison to Huang et al.'s (2020)^8^ study in China, which reported 63% of patients. The reason for this is yet unknown, but it can be due to the immunological response to the virus that changes as it expands to other countries or due to the differences between the studied populations^16^.**

Lymphocyte deficiency in COVID-19 is multi-focal in origin and is a sign of severe disease and prolonged duration of hospitalization. The following mechanisms may explain the cause of lymphopenia^17^. ACE-2 receptor is expressed by lymphocytes, predisposing them to be direct virus target sites. The virus attaches to and attacks lymphocytes, destroying them in the process^17^. Secondly, the virus may cause the destruction of lymphatic organs such as thymus and spleen, predisposing to reduced lymphocyte production. Thirdly, cytokines like tumor necrosis factor-alpha (TNF-α) and interleukin (IL)-6 may get disordered, causing lymphocytes to undergo apoptosis. Finally, the proliferation of lymphocytes may be suppressed in critical COVID-19 patients due to increased metabolic parameters such as lactic acid, which causes hyper-lactic acidemia^17^. Treatment modalities of COVID-19 may further cause lymphopenia. Treatment with glucocorticoid causes apoptosis and migration of lymphocytes from peripheral blood^13,18^, hence contributing to a lymphopenic picture.

## 4. Cytokine Storm

**Figure 2:**
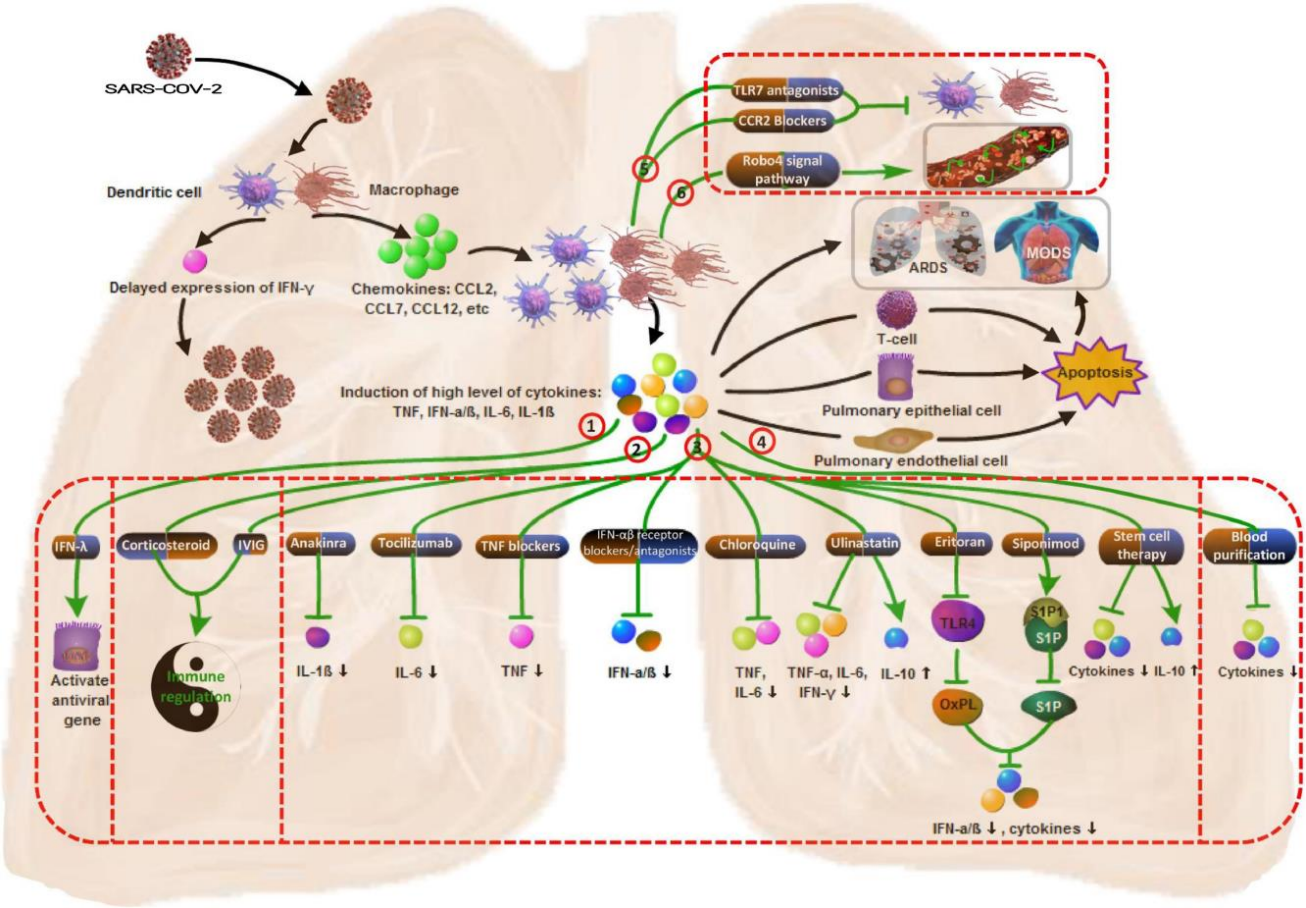
Cytokine storm mechanism Reproduced from Qing et al.^20^ with permission. “Elsevier hereby grants permission to make all its COVID-19-related research that is available on the COVID-19 resource centre - including this research content - immediately available in PubMed Central and other publicly funded repositories, such as the WHO COVID database with rights for unrestricted research re-use and analyses in any form or by any means with acknowledgment of the original source”.

SARS-COV-2 causes the excess formation of pro-inflammatory cytokines, leading to a phenomenon referred to as cytokine storm^19^. A cytokine storm causes vascular hyper-permeability, acute respiratory distress syndrome (ARDS), and even organ failures. It can be fatal if there is excess overproduction of cytokines^20^. The exact process of cytokine storm is illustrated in Figure 2.**

Cytokine release syndrome (CRS) is usually related to immunity disorders that originate from the focal infected area and spreads throughout the body^21,22^***. ***In COVID-19 patients, the cytokine storm can lead to death due to ARDS. It can lead to hypoxia, lung injury, fever, and arrhythmic heart in severe cases^23^. In critically ill patients, organ damage and extra-pulmonary effects of COVID-19 can be attributed to cytokine storm^23^.

IL-6 levels drastically increase in COVID-19 infections due to tissue injuries, increased hematopoiesis, and immune reactions^24^. IL-6 is the basic mediator of virulence in cytokine storms^22,25^; hence the high level of IL-6 is a sign of severe SARS-CoV-2 infection. Diabetic patients might be more vulnerable to cytokine storm due to unwarranted amounts of IL-6 production. Hence, diabetic COVID-19 patients should be dealt with care, as they could be at greater risk of organ failure and development of ARDS^26^.

## 5. Treatment of cytokine storm

Cytokine storm is now recognized as the main cause of many fatalities due to COVID-19. Medical management of cytokine storm is thus essential towards the management of COVID-19 and prevention of further deterioration. Treatment modalities primarily target IL-6, TNF, and IL-1β^27^.

### 5.1. Metronidazole

Metronidazole is a biocidal agent characterized as a redox-active agent that decreases cytokines levels and targets IL-1 beta, IL-6, IL-8, IL-12, and TNF alpha^28^. Metronidazole also has the potential to reduce reactive oxygen species (ROS) produced by neutrophils during inflammation^29^ (Table 2).

**Table 2:** Metronidazole effects on cytokine levels in COVID-19 infection Reproduced from Gharebaghi et al. (2020)^28^ with permission.

COVID-19	Metronidazole
↑IL8	↓IL8
↑IL6	↓IL6
↑IL1B	↓IL1B
↑TNFα	↓TNFα
↑CRP	↓CRP
↑IL12	↓IL12
↑IFNγ	↓IFNγ
↑Neutrophils	↓Neutrophils
↓Lymphocytes	↑Lymphocytes lymphoproliferative properties

### 5.2. Tocilizumab

Tocilizumab is an antagonist of IL-6 and attaches to and blocks signaling of both soluble and membrane-bound IL-6 receptors^21,22^. Tocilizumab serves as an effective treatment choice for patients with severe cytokine storm reactions in COVID-19^25,30^. Le et al. (2017)^31^ clinical trial with intravenous tocilizumab in patients presenting with cytokine storm showed a 69% success rate^31^***.*** US Food and Drug Administration (FDA) thus validated tocilizumab for its application in severe cytokine storm treatment^25^***.***

**Figure 3:**
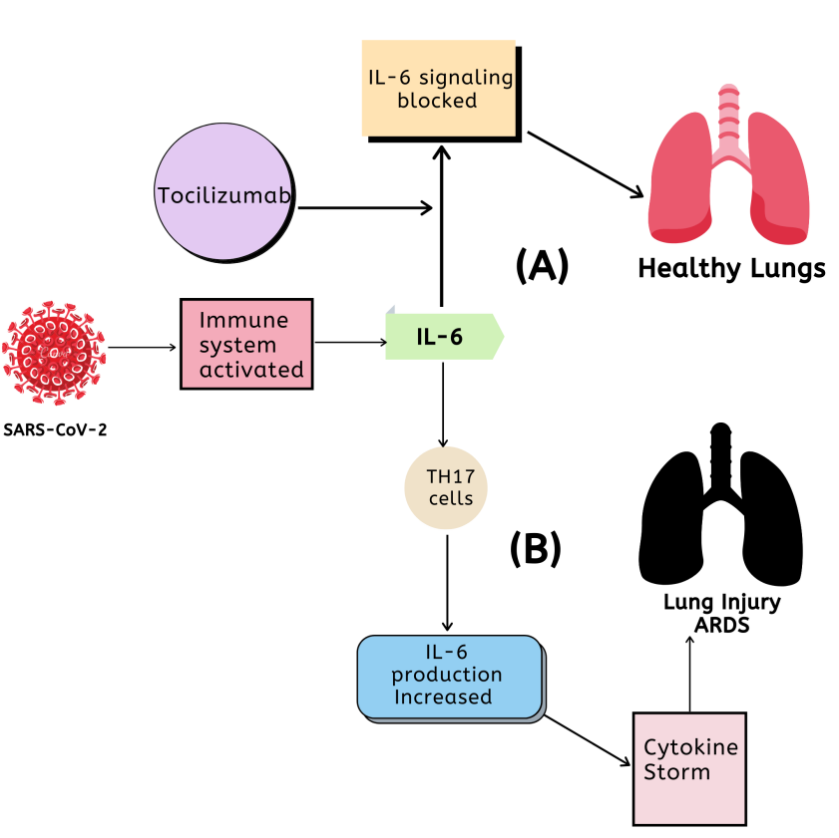
Effect of tocilizumab in subduing the cytokine storm Adapted from^32,33^ with permission. **A.** Tocilizumab blocks IL-6 signaling thus suppressing cytokine storm; **B.** IL-6 activates TH17 (T-helper 17) cells which then increases the IL-6 production. IL-6 occupies a major part in cytokine storm and hence resulting in lung injury and ARDS.

Figure 3 illustrates how tocilizumab could effectively subdue the cytokine storm by inhibiting IL-6 signaling.

### 5.3. Ulinastatin

Ulinastatin is a glycoprotein that functions by producing an anti-inflammatory response. A meta-analysis by Zhang et al. (2019)^34^ indicated that ulinastatin proved significantly helpful in treating ARDS^34^. Ulinastatin decreases pro-inflammatory cytokines levels (IL-6, IFN-γ, TNF-alpha) and can show promising potential as a treatment option for COVID-19^35^ (Table 3).

**Table 3:** Effect of ulinastatin on cytokine levels Adapted from^35,36^ with permission.

Pro-inflammatory cytokines	Ulinastatin
IFN-γ	Decreases^35,36^
TNF-α	Decreases^35,36^
IL-6	Decreases^35,36^
Anti-inflammatory cytokines	Ulinastatin
IL-10	Increases^35,36^

## 6. Coagulation Parameters

Coagulation disorders pose a serious threat to COVID-19 patients, if not appropriately managed, with excessive coagulation leading to thrombosis, as seen in hospitalized patients^37^. Thrombosis is fatal for COVID-19 patients if not promptly addressed. The sudden and rapid increase in D-dimer levels contributes to more severe COVID-19 infection^38^. The coagulation complications of COVID-19 are somewhat analogous to SARS. Deep vein thrombosis and pulmonary embolism were also found to be associated with deaths due to SARS-CoV-1^39^.

A retrospective performed by Tang et al. (2020)^6^ on 183 participants to elucidate the coagulation profile of COVID-19 patients, presented that fibrinogen degradation product (FDP) level was higher with increased prothrombin time (PT) in non-survivors as compared to the survivors. D-dimer levels were also found to be high in non-survivors. The standard of disseminated intravascular coagulation (DIC) was met by 71.4% of the non-survivors^6^, while 0.6% of the survivors also reached the criteria of DIC^6^. Wu et al. (2020)^40^ agrees with these findings in their study on 201 COVID-19 patients admitted to a hospital in Wuhan. The study found that in patients infected with SARS-CoV-2, coagulation disorders, high D-dimer levels and longer PT increased the chances of ARDS^40^.

Tang et al. (2020)^6^ extensively discussed the significance of high D-dimer levels. However, the authors did not allude to the role of lupus anticoagulant (LAC) on thrombosis. Harzallah et al. (2020)^37^ study of 56 SARS-CoV-2 infected patients in France found that 45% of them were positive for LAC, which is a prothrombotic antibody that predisposes to increased thrombosis^37^.

A study on the hematological presentations in 157 patients with SARS attributed by SARS-CoV-1, noted four disseminated intravascular coagulation (DIC) cases^41^. All those patients showed increased D-dimer levels and thrombocytopenia. Activated partial thromboplastin time (aPTT) was also prolonged. Prothrombin time was more prolonged in patients in ICU as compared to patients in the general ward^41^. These findings give weight to Tang et al. (2020)^6^ study findings of DIC and allude to a higher virulence potential of SARS-COV-2 due to a higher DIC rate (71%) in SARS-CoV-2 infected patients^6^.

**Figure 4:**
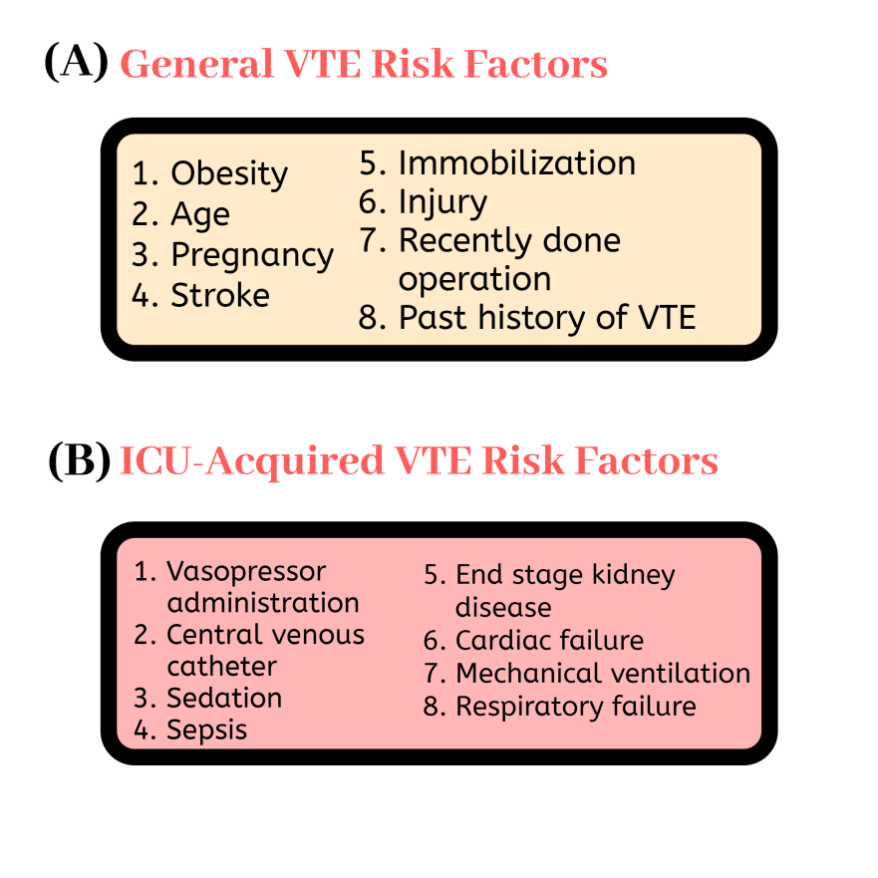
General and ICU acquired venous thromboembolism risk factors Adapted from Minet et al. (2015)^42^ with permission.

The risk of venous thromboembolism (VTE) is substantially increased in critically ill patients of COVID-19^38^. An article by Minet et al. (2015)^42^, describes general and ICU specific risk factors for VTE, as illustrated in Figure 4.

Moreover, pre-existing conditions in COVID-19 patients should also be taken into account while evaluating the coagulation profile. It should be noted that SARS-CoV-2 infected patients with underlying cardiac injury presented with greater coagulopathies than patients without underlying conditions^43^.

## 7. Thrombophilic complications in pregnant women

While discussing coagulation parameters in COVID-19 infection, thrombotic complications in a pregnant woman should not be neglected. Pregnancy substantially increases the risk of hyper-coagulability and women with primary or secondary thrombophilia are more prone to pregnancy complications^44^. Therefore, the management of hyper-coagulability in a pregnant woman infected with COVID-19 needs special attention, since COVID-19 can aggravate the thrombotic complications. Moreover, pregnant women with reproductive failure are at greater risk of severe SARS-COV-2 disease, as there are chances of developing acquired thrombophilia^45^. Low molecular weight heparin (LMWH) is a good option as an anticoagulant to treat pregnant women with reproductive failures^45^. However, further research on the thrombotic complications and its treatment options in pregnant women infected with SARS-CoV-2 is the need of the hour.

## 8. Pre-existing Thrombophilic Disorders And COVID-19

Thrombophilia is described as a state of hyper-coagulability and comprises two types, i.e., primary (hereditary) and secondary (acquired)^46^.

### 8.1. Factor V Leiden mutation

Factor V Leiden mutation is the most common hereditary thrombophilia^47^. In Leiden variants, pulmonary embolism (PE) is less common than deep vein thrombosis (DVT), mainly manifesting in the legs. VTE risk is also increased in Factor V Leiden patients^48^. A study shows an elevated risk of PE and DVT in COVID-19 patients and alludes to a direct correlation with the severity of the disease^49^. Therefore, patients with mutated factor V Leiden are more susceptible to COVID-19, since this factor potentially increases the risk for VTE.

### 8.2. Antiphospholipid syndrome

Antiphospholipid syndrome (APS) is a vasculopathy, as well as a cause of thrombophilia. Renal complications of the antiphospholipid syndrome include nephropathy, renal artery stenosis, and end-stage kidney disease^50^. In primary APS, the triggering of the mTOR (mammalian target of rapamycin) pathway in vascular endothelium results in vascular lesions^51^. The lesions are also present in carotid, coronary, and mesenteric arteries that may lead to ischemia, myocardial infarction, and stroke^52^. Based on the fact that underlying cardiovascular disorders in patients with SARS-CoV-2 infection, multiplies the severity of disease^53^, APS patients with vascular lesions may experience a more severe form of COVID-19, with a higher mortality rate. Anticardiolipin antibody (ACA) and lupus anticoagulant (LPA) are present in APS and thus, increases the chances of thrombosis and thrombocytopenia^54^. According to a study, 45% of COVID-19 patients out of 56 were LAC (lupus anticoagulant) positive^37^. From this, it can be inferred that patients of the antiphospholipid syndrome could be more susceptible to COVID-19, due to increased thrombotic complications.

### 8.3. Nephrotic syndrome

The nephrotic syndrome, which is a glomerular disease characterized by edema, proteinuria, hyperlipidemia, and hypoalbuminemia^55^, causes thrombophilia. According to a recent study, out of 10461 patients with nephrotic syndrome, 15 had acute cerebral damage, and D-dimer levels were increased in 13 out of those 15 patients. Coagulopathy was observed in 9 out of the 15 patients^56^. In a study of 100 nephrotic syndrome patients, it was observed that 53 out of 100 had elevated D-dimer levels^57^. A cohort study involving 201 patients showed that coagulation disorders, elevated D-dimer levels, and longer PT amplifies the chances of ARDS in SARS-CoV-2 infected patients^40^. Hence, patients suffering from nephrotic syndrome and COVID-19 have poor outcomes and are at increased possibility of having critical COVID-19 infection. Thus, it can be inferred that pre-existing nephrotic syndrome may increase the risk of hyper-coagulability in COVID-19 patients.

Regular testing for pre-existing thrombophilic conditions in COVID-19 patients is not usually suggested. However, depending on the clinical feature of patients, testing for thrombophilia should not be delayed, since unmanaged thrombophilia can lead to fatal complications^58^.

## 9. Conclusion

· COVID-19 is now considered more as a systemic infection rather than the common flu.

· Hemodynamics of COVID-19 disease gives an in-depth view of the pathophysiology of the disease and possible management and treatment options.

· Hemodynamic picture of patients infected with SARS-CoV-2 is greatly dependent on the severity of the disease.

· Lymphopenia, thrombocytopenia, eosinophilia, neutrophilia, and leucopenia in general are some findings seen in most of the COVID-19 patients especially in ICU ones.

· Hematologic manifestations of COVID-19 patients resemble in many aspects to those observed in SARS and MERS patients.

· A significant number of deaths due to COVID-19 infection can be attributed to cytokine storm and cytokine release syndrome.

· IL-6 signaling plays a drastic part in the cytokine storm. Therefore, drugs inhibiting cytokine storm can help in treating the infection.

· Metronidazole, tocilizumab, and ulinastatin potentially decrease cytokine levels and thus could suppress cytokine storm.

· Non-survivors of SARS-CoV-2 disease showed higher D-dimer levels, prothrombin time, and fibrinogen degradation products, if compared to survivors.

· Pre-existing thrombophilic conditions, including both hereditary and acquired thrombophilia, worsens the COVID-19 disease and are a poor prognostic marker for the disease.

· The inverse may also be true, i.e., COVID-19 can exacerbate thrombophilia, due to a rise in D-dimer levels and longer PT.

· In itself, the disease actuates a hyper-coagulable state and is linked to a higher risk for VTE due to excessive inflammation, hypoxia, and immobilization, particularly in severely ill COVID-19 patients.

· Management of coagulation-related compli-cations in pregnant women with SARS-CoV-2 infection is challenging and further research on this aspect should be undertaken.

· Testing for pre-existing acquired thrombophilia in a COVID-19 patient should only be performed if the clinical presentation suggests so.
